# Inhibition of Astrocytic Histamine *N*-Methyltransferase as a Possible Target for the Treatment of Alzheimer’s Disease

**DOI:** 10.3390/biom11101408

**Published:** 2021-09-26

**Authors:** Cecilia Flores-Clemente, María Inés Nicolás-Vázquez, Elvia Mera Jiménez, Maricarmen Hernández-Rodríguez

**Affiliations:** 1 Laboratorio de Cultivo Celular, Sección de Posgrado e Investigación, Escuela Superior de Medicina, Instituto Politécnico Nacional, Ciudad de México 11340, Mexico; cecifloresclemente1@gmail.com (C.F.-C.); elviamj@gmail.com (E.M.J.); 2 Departamento de Ciencias Químicas, Facultad de Estudios Superiores Cuautitlán Campo 1, Universidad Nacional Autónoma de México, Cuautitlán Izcalli 54714, Mexico; nicovain@yahoo.com.mx

**Keywords:** Alzheimer’s disease, neurotransmitters, histamine, histamine *N*-methyltransferase-HNMT, astrocytes

## Abstract

Alzheimer’s disease (AD) represents the principal cause of dementia among the elderly. Great efforts have been established to understand the physiopathology of AD. Changes in neurotransmitter systems in patients with AD, including cholinergic, GABAergic, serotoninergic, noradrenergic, and histaminergic changes have been reported. Interestingly, changes in the histaminergic system have been related to cognitive impairment in AD patients. The principal pathological changes in the brains of AD patients, related to the histaminergic system, are neurofibrillary degeneration of the tuberomammillary nucleus, the main source of histamine in the brain, low histamine levels, and altered signaling of its receptors. The increase of histamine levels can be achieved by inhibiting its degrading enzyme, histamine *N*-methyltransferase (HNMT), a cytoplasmatic enzyme located in astrocytes. Thus, increasing histamine levels could be employed in AD patients as co-therapy due to their effects on cognitive functions, neuroplasticity, neuronal survival, neurogenesis, and the degradation of amyloid beta (Aβ) peptides. In this sense, the evaluation of the impact of HNMT inhibitors on animal models of AD would be interesting, consequently highlighting its relevance.

## 1. Introduction

Nowadays, Alzheimer’s disease (AD) represents a disorder with no permanent cure. The main clinical manifestations are related to learning and memory disabilities and other cognitive symptoms that impair independence and quality of life [1]. AD is the principal cause of dementia among the elderly. Although several efforts have been made to understand the physiopathology of AD, the exact causes are still not completely understood [2]. The study of AD has been focused on two principal histopathological hallmarks: amyloid β (Aβ) plaque deposits and intracellular neurofibrillary tangles (NFTs) in the brain [3]. According to these, several compounds have been designed to avoid Aβ production or aggregation. However, results in clinical assays have not been promising [4]. In this sense, the search for novel targets results in great interest. Recent studies have been shown changes in several neurotransmitters. The principal neurotransmitter systems altered in patients with AD are cholinergic and glutamatergic systems, which are especially affected by neurodegeneration, which in turn, allowed for the development of acetylcholinesterase (AChE) inhibitors and antagonists of *N*-methyl-d-aspartate (NMDA) receptors, the main drugs employed to treat AD [5]. However, a great number of studies have described changes in other neurotransmitters in the brains of AD patients, including the serotoninergic [6], the noradrenergic [7], and the histaminergic [8] systems which could be due to global affectation of the brain while AD progresses.

Brain histaminergic system results are of particular interest as a potential target to modify the cognitive symptoms of AD [8]. In this sense, enhancing histaminergic neurotransmission in AD patients could result in beneficial effects such as improvement of cognitive symptoms and neuroplasticity [9] increase in the degradation of extracellular Aβ insoluble plaques [10], lowering Aβ pathology [11] and increase neurogenesis [12].

In this sense, it has been demonstrated that the regulation of histamine concentration in the extracellular space of the central nervous system (CNS) is regulated by histamine *N*-methyltransferase (HNMT), located mainly in astrocytes, that degrades histamine to tele-methylhistamine [13]. Thus, the employment of HNMT inhibitors to increase histamine levels could represent a beneficial approach to enhance cognitive abilities in AD patients.

## 2. General Aspects of AD

AD is a neurodegenerative disorder that represents the major cause of dementia. Clinically, AD is described by changes in cognitive functions and impairment in the ability to achieve personal daily activities [14,15,16].

Traditionally, the study of AD has been focused on two major histopathological findings: senile plaques composed principally by Aβ aggregates and NFTs composed of hyperphosphorylated tau protein [17].

### 2.1. Aβ as a Neurotoxic Specie

**Figure 1:**
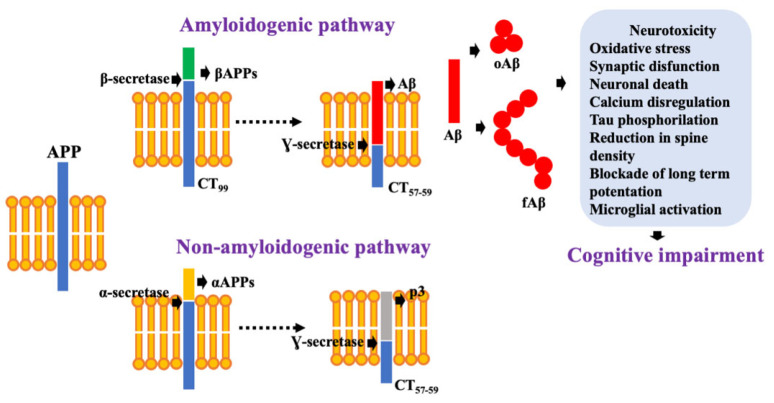
Aβ production following the amyloidogenic pathway. Once Aβ is released it tends to establish interaction with other monomers to form oligomeric (oAβ) and fibrillar species (fAβ), which are highly neurotoxic.

Aβ is a 39–43 residue amyloidogenic peptide released after the cleavage of amyloid precursor protein (APP) [18]. APP is a glycoprotein processed by several proteases, following two processes that compete for the same part of the protein [19]. In the most common pathway, APP is cleaved sequentially by α-secretase and γ-secretase. This pathway is known as the non-amyloidogenic due to the prevention of Aβ peptide release (Figure 1) [20]. However, APP is processed in AD by β-secretase (BACE1) and γ-secretase, following the amyloidogenic pathway, which favors Aβ peptide release (Figure 1). Once Aβ is released, it interacts with other monomers resulting in the formation of soluble oligomers (oAβ) and insoluble fibrils (fAβ) [19,20].

Significant evidence has pointed oAβ as the most neurotoxic form of Aβ [21]. oAβ occurs early, before senile plaques in the AD brain [22,23].

Recently, it was demonstrated that oAβ could bind to several surface receptors present in neurons and glial cells favoring synaptic disfunction such as NMDA receptor and GABAergic receptors, among others [24]. Additionally, oAβ binds to the a7nAChR with high affinity [25]. The a7nAChR mediates Aβ-induced tau phosphorylation via ERK and JNK. In addition, Aβ can interact with phospholipids of the cell membrane and create pores and thus neuronal damage [26].

In contrast, fAβ represents the main component of senile plaques. In senile plaques, fAβ deposits are surrounded by synaptic loss, activated microglia, and reactive astrocytes [27]. The development of an oxidative stress state which in turn results in neurotoxicity, has been related to fAβ. Furthermore, reactive oxygen species enhance Aβ levels and accumulate, resulting in the potentiation of neuronal damage [28].

### 2.2. NFTs Correlate with Cognitive Impairment

NFTs represents the kind of intracellular aggregates which are widely found in the hippocampus, the entorhinal cortex, and the basal forebrain, these being brain zones particularly affected by neurodegeneration [29,30]. NFTs are composed of hyperphosphorylated and abnormally folded tau protein, thus, lacking its function to stabilize microtubules in the axon [31]. Interestingly, NFTs have been positively correlated with cognitive impairment [32], probably due to synaptic impairment in AD brains. Synaptic damage induced by NFTs results from both impaired axonal transport and impaired synaptic transmission in dendritic spines [33]. Interestingly, it has been postulated that NFTs could be a consequence of Aβ increase [34].

## 3. Dysregulation of the Neurotransmission Systems Involved in AD

Several studies of post-mortem AD brains have shown changes in monoamine transmitter systems including serotonin [6], noradrenalin [7], histamine [8], and ACh [35] which tend to show early and severe damage. Low monoamine levels have been found, which precede the loss of its producing neurons [36]. For this reason, significant efforts have been made to determine the relationship between neurotransmitters dysfunction and AD pathogenesis [37].

### 3.1. Cholinergic System

The nucleus basalis of Meynert (NBM) represents the primary source of cholinergic innervation in the CNS. Cholinergic neurons produce acetylcholine (ACh) in synaptic terminations by choline acetyltransferase (CAT), which employs acetyl coenzyme A and choline. ACh exerts its effects by binding to muscarinic (M1 to M5) and nicotinic receptors. Termination of acetylcholine action occurs when ACh is degraded by acetylcholinesterase (AChE) into choline and acetate [38].

Post-mortem studies allowed for correlating the impairment of cortical cholinergic innervation with the presence of NFTs in the NBM [39]. Additionally, the low activity of CAT has been associated with a high number of senile plaques in the post-mortem brains of AD patients [40]. Lowering the cholinergic system produces an increase of both Aβ deposition and NFTs which contribute to cognitive impairment [41]. Consequently, the importance of pathological changes in the cholinergic system of AD patients is reinforced by the fact that the principal strategy in the treatment of AD patients is the increase of availability of ACh by AChE inhibitors (donepezil, rivastigmine, and galantamine) [42].

### 3.2. Glutamate and NMDA Receptors

Glutamate is the principal excitatory neurotransmitter in CNS. Glutamate receptors are mainly ligand-gated ionotropic receptors and play fundamental roles in synaptic plasticity, learning, and memory [43]. One subgroup of glutamate receptors are *N*-methyl-d-aspartate (NMDA) receptors. NMDA receptors are essential for neuronal survival by activating the neuronal survival pathway [44].

In the brains of AD patients, the principal alteration associated to glutamate signaling is the chronic hyperactivation of NMDA receptors which results in excessive Ca^2+^ entry to the postsynaptic neuron [45]. Thus, the pathological increase in signaling related to Ca^2+^ impairs synaptic function, leading to neuronal cell death. Interestingly, neuronal damage is correlated with clinical deterioration in cognition/memory seen in AD patients. Thus, the findings allow for the design and evaluation of the unique NMDAR antagonist employed to treat AD and memantine [46].

### 3.3. Serotonergic System

According to their mechanism of action, serotonin exerts its effects by binding to 16 types of serotonin receptors, which belongs to seven sub-families, 5-HT1 to 5-HT7 [47].

Several studies have correlated cognitive deficits, impairments in learning, and memory decline with 5-HT and its receptors [48]. In the brains of AD patients, an increase of 5-HT1A receptor density has been observed, which positively correlates with cognitive impairment [49]. Similar, 5-HT2 receptor has been closely related to cognitive dysfunction. In this way, Blin et al. reported a critical reduction in the 5-HT2 receptor binding in the cerebral cortex of AD patients compared to healthy controls [50], suggesting a correlation between neocortical 5-HT2A expression and cognitive decline in AD patients.

### 3.4. Noradrenergic System

Norepinephrine (NE) is released by the locus coeruleus (LC) in the CNS and regulates many number of cellular processes by interacting with its receptors. Degeneration of LC by the increase of NFTs represents the principal pathological change in the adrenergic system in AD patients [51]. The noradrenergic innervation that reaches the cerebral vasculature optimizes the delivery of oxygen which, when it deteriorates, decreases the oxygen supply capacity that can also contribute to the pathogenesis of AD [52].

Deposits of hyperphosphorylated tau in the hippocampus and noradrenergic axonal degeneration in the brains of AD patients is related to cognitive impairment, which is explained by the importance of NE in long-term potentiation and synaptic plasticity [53].

### 3.5. Histaminergic System

Histaminergic neuron bodies are located in the tuberomammillary nucleus (TMN) in the posterior hypothalamic region between the mammillary body and the optic chiasm. TMN nucleus receives significant input from the limbic areas and projects diffusely to large parts of the central nervous system [54,55,56]. Histidine decarboxylase (HDC) is the enzyme responsible to synthesize histamine from l-histidine [57]. Histamine exerts its effects by histamine receptors 1, 2, 3, and 4 (HR1 to HR4, all of which are G protein-coupled) [58].

**Figure 2:**
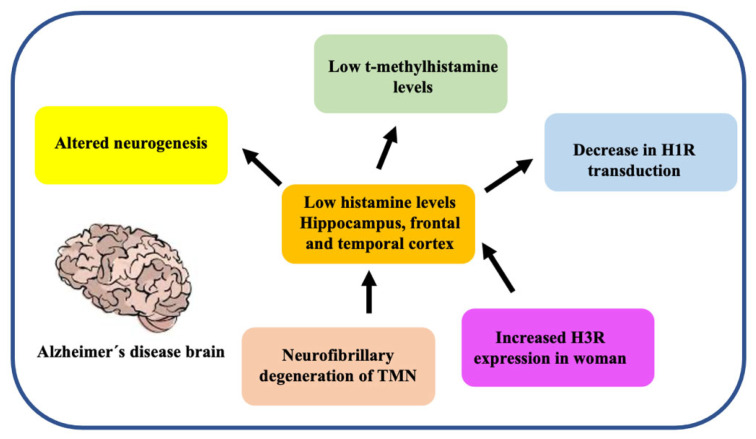
Principal changes in the histaminergic system in the brain of AD patients. AD patients show many alterations related to the histaminergic system such as low brain histamine levels, low H1R expression in both the frontal and temporal cortex, and degeneration induced by neurofibrillary tangles in TMN, the main source of histamine in the brain.

The regulation of histamine levels in the synaptic space occurs predominantly by the action of HNMT that degrades histamine to t-methylhistamine [13]. The histaminergic system involves cognitive functions related to regulating the sleep-wake cycle, sensory and motor functions, energy and endocrine homeostasis, cognition, attention, learning, and memory [53]. All of these modalities are severely affected in AD. In addition, several changes have been reported in the brains of AD patients (Figure 2) [59].

Although there are contradictory findings related to the histaminergic system and AD, decreased histamine levels have been detected in several brain areas of AD patients such as the hippocampus and the temporal cortex [60]. Oh et al. 2019, demonstrated a 62% reduction in TMN neurons, explaining the low levels of histamine in the brains of AD patients [61]. TMN degeneration has been associated with neurofibrillary damage. Thus, explaining the cognitive impairment in AD patients [62].

In addition, in a study conducted to measure t-methylhistamine in cerebrospinal fluid, as a marker of histaminergic system activity, shown lower levels of t-methylhistamine were found in CSF of AD patients compared to control subjects [63].

It has been shown that the general levels of HDC mRNA in the TMN remained practically unchanged in patients with AD regardless of gender, except for a decrease in HDC mRNA in the medial part of the TMN [64]. Additional to low histamine levels in the brains of AD patients, an increased expression of H3R and HNMT in females was observed [64].

Additionally, another study showed a decrease in ligand binding to H1R in AD patients compared to normal subjects, especially in frontal and temporal regions, where they found decreased expression of H1R, evidenced by positron emission tomography [65]. Interestingly, H1R knockout mice exhibit a high degree of alterations in learning and memory, thus impairing working memory [66].

H1R density in the frontal and temporal regions of AD patients has been documented to be decreased compared to healthy subjects of the same age. It is suggested that this could also be associated with changes in histamine levels and H1R expression, sensitivity, and/or H1R transduction [67].

Recently, neurogenesis in hippocampal formation was reported to be altered in H1R-knockout mice. Significantly, they showed a reduction in newborn neurons, but there was no change related to the differentiation of progenitor neurons into neuronal and glial lineages [12]. Likewise, increased levels of dopamine and lower production of its dihydro phenylacetic acid metabolite were recorded in the amygdala of H1R-deficient mice. This could be explained by the greater immunoreactivity of tyrosine hydroxylase (greater synthesis of catecholamines epinephrine, NE, and dopamine) in the anterior basolateral, ventral basolateral, and cortical nuclei of the amygdala [8]. Furthermore, due to the decrease in histamine synthesis in HDC-deficient mice, it was showed differential effects in ACh levels. In contrast, ACh levels increase in the frontal cortex, ACh levels lower in the neostriatum [68].

A relation between cognitive impairment in AD patients and decreased histaminergic activity has been established based on the activation of septohippocampal GABAergic neurons elicited by histamine through both direct and indirect (cholinergic) mechanisms, which are related to cognition and memory [69].

It is worth mentioning that there are few studies on the role played by histamine H4R receptors in AD. H4R activation has been related to the regulation of inflammatory responses and migration of microglial cells. In contrast, H4R activation can lower microglial activation after their exposition to lipopolysaccharide [70].

Given the significant changes in the histaminergic system in the brains of AD and the beneficial effects exhibited by increasing histamine levels by H3R receptors, the search for novel mechanisms to increase brain levels of histamine are needed.

## 4. Histamine in the Brain

### 4.1. Histaminergic System in the CNS

Histaminergic neurons have a large soma 20–30 µm in diameter, with two to three large, subdivided dendrites that overlap the dendrites of other histaminergic neurons. They have a large cytoplasm with a large nucleus, a prominent dark nucleolus, a well-developed Golgi apparatus, and abundant mitochondria. Varicose axons arise primarily from a thick dendrite and not the neuron soma [71,72].

The afferents of the TMN come from different regions such as the cortex and preoptic area of the hypothalamus (glutamatergic fibers and GABAergic fibers), basal forebrain, middle septum, diagonal band of Broca, NBM and innominate substance (cholinergic fibers), and the locus coeruleus (noradrenergic fibers) [73,74,75,76]. The TMN sends axonal projections to different brain areas through two ascending pathways and one descending pathway. The first ascending pathway travels from the ventral surface of the median eminence to the hypothalamus, the diagonal band, the septal area, and the olfactory bulb, hippocampus, and cortex. The second arises from the dorsal region of the TMN, reaches the third ventricle to the thalamus, the basal ganglia, hippocampus, amygdala, and cortex. The descending pathway runs from the medial longitudinal bundle to the brainstem and spinal cord. There appears to be no topological correlation between the bodies of the TMN neurons and the projection of the axons. Histaminergic fibers have been observed to cross extensively, and several neurons branch to more than one initial pathway [53,57].

**Figure 3:**
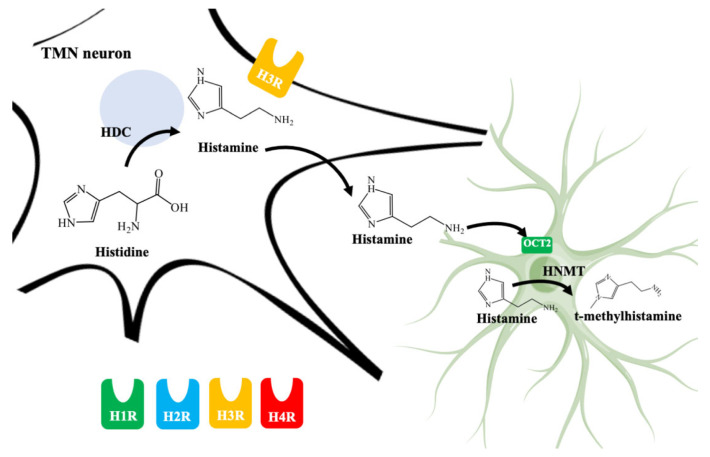
Schematic illustration of histamine synthesis, degradation, and types of histamine receptors. Histamine is synthesized by histidine decarboxylase (HDC) in neurons from the tuberomammillary nucleus (TMN). Histamine could interact with histamine receptors 1 to 4 (H1R, H2R, H3R, and H4R). Importantly, H3R are located presynaptically and regulates histamine release. The effect of histamine is ended by recapture in astrocytes mainly by organic cation transporter 2 (OCT2) and subsequent degradation by histamine *N*-methyltransferase (HNMT) which is located in the cytosol.

As it can be seen in Figure 3, histamine is synthesized by HDC from l-histidine [58]. HDC is expressed both in the cell body, axonal, and terminal neuronal varicosities. One of the limiting factors in the synthesis is the bioavailability of the substrate [77].

It has been shown that histamine, as with other monoamines, is transported from the cytoplasm of presynaptic nerves, through an electrochemical proton gradient generated by vacuolar H^+^ adenosine triphosphatase, to secretory vesicles in neurons by the vesicular monoamine transporter 2 (VMAT-2) [78]. Histamine is released in the soma and especially in the axonal varicosities of neurons after the arrival of action potentials [79,80]. Both the synthesis and the release of monoamine are regulated by the feedback of the activation of histamine H3 (H3R) autoreceptors [81].

### 4.2. Histamine Receptors

**Figure 4:**
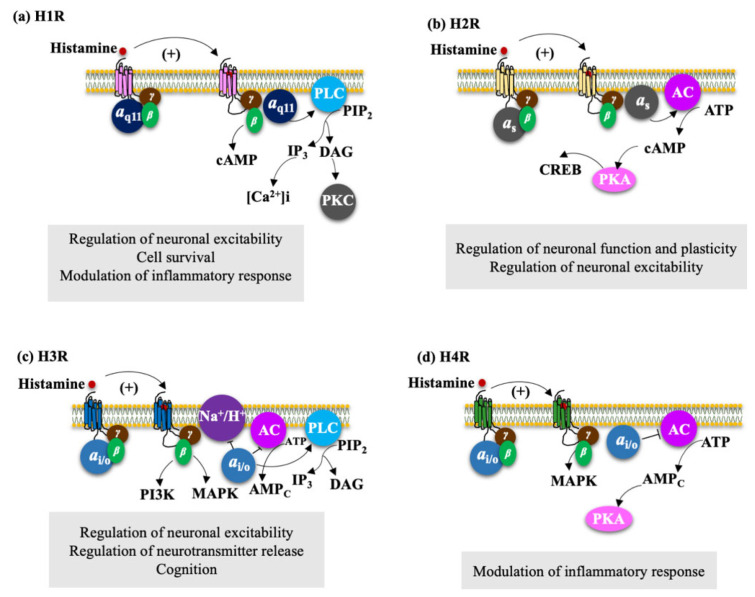
Signaling pathways triggered by histamine receptor activation. Activation of the H1R-H4R receptors, figures (**a**–**d**). The H1R receptor is widely expressed in the hippocampus, cholinergic and aminergic brain stem nuclei, thalamus, and cortex. H2R is located in the basal ganglia, amygdala, hippocampus, and brain cortex. High H3R densities have been demonstrated in the anterior cerebral cortex, hippocampus, amygdala, striatum, olfactory tubercle, cerebellum, substantia nigra, and brain stem. Finally, expression of H4R mRNA has been reported in the amygdala, cerebellum, corpus callosum, frontal cortex, hippocampus, and thalamus.

Histamine exerts its actions by its binding with four different G protein-coupled receptors (GPCRs) (H1R, H2R, H3R, and H4R) [82]. Each receptor has its characteristics and activates signaling pathways (Figure 4).

#### 4.2.1. H1R Regulate Neuronal Excitability, Cell Survival, and Modulation of Inflammatory Response

H1R is widely expressed in the CNS and peripheral nervous system (PNS) with considerable variations between species [83,84]. High H1R densities are expressed in the hippocampus, cholinergic and aminergic brain stem nuclei, thalamus, and cortex, areas involved in neuroendocrine processes, behavior, and food intake [85]. H1R is highly expressed in both neurons and astrocytes [86]. In astrocytes, H1R expression can be positively and selectively regulated by histamine [87].

H1R receptor binds to Gαq/11 proteins [88]. Its activation triggers phospholipase C (PLC) signaling (Figure 4a), which produces 1,2-diacylglycerol (DAG) and inositol-1,4,5 -triphosphate (IP3), leading to the activation of protein kinase C (PKC) and consequently to the catalysis of phosphorylation of Serine-Threonine residues (Ser/Thr) of several mediators, resulting in the release of calcium ions (Ca^2+^) of intracellular stores, causing an increase in the concentration of this ion and the activation of the Na^+^/Ca^2+^ exchanger [89,90].

H1R activation excites brain stem neurons [91], septum [91], thalamus [92], amygdala, hippocampus [93], and olfactory bulb [94]. However, receptor activation can also inhibit the firing of hippocampal pyramidal neurons by activating K^+^ channels by increasing the concentration of intracellular calcium [Ca^2+^]i [94]. In astrocytes, H1R activation inhibits the release of pro-inflammatory factors such as tumor necrosis factor-alpha (TNF-α) and interleukin-1β (IL-1β) molecules, which are important for cell survival and suppression of the inflammatory response [87].

#### 4.2.2. H2R Regulates Neuronal Plasticity and Neuronal Excitability

H2R is widely expressed in CNS and PNS. In CNS, high H2R receptor densities are located in the basal ganglia, amygdala, hippocampus, and cortex [95]. Furthermore, its high expression in neurons [96] and astrocytes has been demonstrated [87]. Histamine deficiency downregulates H2R expression, but not H1R in HDC knockout mice [97]. The receptor is mainly coupled to the Gαs protein (Figure 4b). Its activation stimulates adenylyl cyclase (AC), thus increasing the concentration of cyclic adenosine monophosphate (cAMP) [98], which in turn activates the protein kinase A (PKA) and the transcription of cAMP response binding element (CREB), important regulatory molecules in neuronal plasticity and function [56]. Ca^2+^-activated potassium (K^+^) (KCa) channel block, dependent on PKA phosphorylation, promotes neuronal excitability [99].

#### 4.2.3. H3R Regulate Neuronal Excitability, Neurotransmitter Release, and Cognition

H3R was identified by Arrang et al. in 1983, and the human H3R was cloned by Lovenberg et al. in 1999 [100]. Unlike the H1R and H2R receptors, the H3R has isoforms originated by alternative splicing of messenger ribonucleic acid (mRNA), each with different pharmacological properties and distribution in the CNS. The most abundant in the brain is the 445 amino acid isoforms (H3R445) [56]. In humans, 20 isoforms have been reported, and six of them have shown functionality when expressed heterologous (hH3R453, hH3R445, hH3R431, hH3R415, hH3R373, hH3R365) [45].

High H3R densities have been observed in the anterior cerebral cortex, hippocampus, amygdala, cerebellum, and substantia nigra [60,101]. Furthermore, their presence in both neurons and astrocytes have been identified [87]. H3R is expressed as an autoreceptor in soma, dendrites, and axons of TMN neurons and its activation reduces cellular excitability and regulates the release and synthesis of histamine [102,103]. Activation of H3R as a presynaptic heteroreceptor regulates the release of other neurotransmitters, including biogenic amines [104,105], GABA [106], glutamate [107,108], and neuropeptides [109]. The receptor binds to Gαi/o proteins, which triggers different intracellular signaling pathways (Figure 4c). Through the Gαi/o subunit, it inhibits AC and the accumulation of cAMP [110,111], which in turn avoids the activation of the CREB, which is related to cognitive functions inhibits the Na^+^/H^+^ exchanger [112], activates the PLC pathway and increases [Ca2+]i from intracellular deposits via inositol- 1,4,5-triphosphate (IP3) (PLC/IP3/[Ca^2+^]i) [113]. Through the βγ subunits, it inhibits the N and P/Q type voltage-gated Ca2+ channels, activates the G-protein-activated input rectifier K^+^ channels (GIRK) [114], activates the phosphorylation of mitogen-activated protein kinase (MAPKs) [115,116] —related to memory improvement in the rat activates phosphatidylinositol 3-kinase (PI3K) which afterward activates protein kinase B that phosphorylates and inhibits glycogen synthase kinase-3β (GSK3β), one of the main brain tau kinases [117], and activates phospholipase A2 (PLA2) that induces the release of arachidonic acid (AA) [118].

Unlike the neuronal receptor, there is currently no evidence that related to Astrocytic H3R, constitutive cyclic AMP signaling activity is functionally coupled to Gi/o-dependent inhibition of adenylate cyclase and modulation of the PKC signaling cascade, MAP kinase, and PI3K/AKT [119].

#### 4.2.4. H4R Modulate the Inflammatory Response

The H4 receptor is found in cerebral blood vessels and microglia, but its expression in neurons is not yet well established [120]. In addition, no expression was observed in mammalian primary astrocytes [87].

The H4R was identified and cloned by different research groups [121,122,123]. It shares similarities with H3R in 40% of its structure, in its coupling to Gαi/o proteins (Figure 4d), resulting in decreased cAMP production by AC, increased Ca2+ mobilization, activation of extracellular signal-regulated kinase (ERK) 1/2 and Akt, and cytoskeletal changes promoting cell survival [121,124]. It is abundantly expressed in bone marrow, cells, and peripheral tissue. In CNS, the immunological detection of H4R does not always correspond to the expression of mRNA so its expression has not yet been clearly described. In humans, H4R mRNA expression has been reported in the amygdala, cerebellum, spinal cord, frontal cortex, hippocampus, and thalamus [125,126].

### 4.3. Astrocytes Are Involved in Histamine Elimination

Astrocytes are most abundant of the glial cells. The main functions of astrocytes include: the elimination of toxic substances, protection from oxidative damage, maintain ionic homeostasis, energy metabolism, elimination of neurotransmitters, neurotrophic activity, and the immune response, thus fulfilling essential protective and restorative functions. After an injury or pathological process which affects the brain, astrocytes undergo morphological changes to increase their metabolic activity [127]. In addition, astrocytes have been shown to support the formation and integrity of the endothelial barrier function known as the blood-brain barrier (BBB) [128].

The role of astrocytic cells in histamine degradation has been described previously [13,56]. As it can be seen in Figure 3, human astrocytes transport histamine dominantly through organics cations transporter 2 and 3 (OCT2, OCT3) and plasma membrane monoamine transporter (PMAT) [129]. Next, histamine is transported into the cytosol and metabolized by HNMT responsible for methylation of the imidazole ring to t-methylhistamine. Therefore, the fluctuation of histamine and t-methylhistamine levels can provide accurate information on histamine turnover and the activity of histaminergic neurons, latter the t-methylhistamine is which is later converted to t-methyl-imidazole acetic acid through monoamine oxidase B (MAO-B) [130]. Histamine degradation by HNMT represents the unique known pathway for the termination of histaminergic neurotransmission in the human CNS [131]. Indeed, HNMT knockout mice showed increased brain levels of histamine, highlighting the contribution of HNMT to histamine inactivation [132].

HNMT is polymorphic due to genetic changes. The HNMT gene, located in chromosome 2q22.1, shows diverse single nucleotide polymorphism (SNPs), and one of these, located in exon 4 C314T, causes the amino acid substitution Thr105Ile [133]. This variant acquires importance due to the lowering of enzymatic HNMT activity [134]. Further genetic studies showed the relation of C314T substitution to other neurological disorders such as schizophrenia [135], attention deficit hyperactivity disorder (ADHD) [136], and migraine [137] but not AD [138]. 

In contrast, a polymorphism at nucleotide 939 in the human HNMT increases both HNMT protein expression and activity. This polymorphism was associate with myasthenia gravis and ADHD [138,139].

Given the relevance of histamine in brain functions, it is worth examining the changes in HNMT expression in neurological disorders. According to these, post-mortem studies showed an increase in HNMT mRNA expression in the prefrontal cortex of female AD patients [64]. Although recent findings suggest a possible relation between HNMT activity and neurological disorders, the impact of changes in HNMT activity in disease onset and progression is yet to be identified.

## 5. Increase Histamine Levels in the Brain as an Opportunity to Develop Novel Treatments for AD Patients

Increasing histamine levels in the CNS can be achieved through two strategies: by H3 receptor antagonist/inverse agonist and by HNMT inhibitors.

### 5.1. H3R Inverse Agonist/Antagonist

Lowering the presynaptic H3R activation by its antagonist/inverse agonist allows for increasing the histamine level by blocking the release mechanism´s negative feedback. Some research groups have focused to develop H3R modulators to alleviate disease-related behavioral patterns in AD patients. In this sense, although not all studies have shown favorable results, some H3R antagonists are important to highlight. In a clinical study, the employment of H3R antagonist (GSK239512) demonstrated improvements in cognitive function in AD patients with mild to moderate clinical manifestations [140].

Experimental studies corroborated that H3R antagonists/inverse agonist molecules such as JNJ-10181457, Thioperamide, Clobenpropit, JNJ-5207852, among others, and can restore cognitive functions in a wide variety of murine amnesia models [8]. Provensi et al. showed that both donepezil and ABT-239 (H3R antagonist) enhance cognitive activities in mice with intact brain histamine systems [141], highlighting the importance of enhancing the histaminergic system in AD patients. However, the reverse transcription-polymerase chain reaction (RT-PCR) analysis has enabled identifying 20 hH3R isoforms, with differences in the amino and carboxyl-termini length, and sequence deletions resulting in a high variability [142]. The complexity of H3R biology makes it difficult for the pharmacological evaluation of H3R antagonist/inverse agonist. Thus, more approaches to increase histamine levels in the brain are needed.

### 5.2. Inhibition of HNMT

Under physiological conditions, normal neurotransmitters levels are maintained by its clearance, which can be achieved by diffusion, transporters, and /or degrading enzymes. Transporters or enzymes are usually located in surrounding neurons or astrocytes [143]. The modification of neurotransmitter clearance systems in the brain allowed for the development of various drugs such as AChE inhibitors, tricyclic antidepressants, and serotonin re-uptake inhibitors to treat brain diseases [144]. This evidence highlights that histamine degrading mechanisms could be a therapeutic approach for developing novel drugs that improve brain functions.

As previously described and shown in Figure 3, HNMT represents the exclusive enzyme that inactivates histamine in the brain. Although HNMT has been localized in synaptosomes, it is reported that HNMT located in astrocytes is 70% more active, highlighting the importance of astrocytes in histamine removal and inactivation [145,146]. In this sense, inhibitors of HNMT have been developed, generally, designed as histamine receptors antagonist or to other proposes. As it can be seen in Table 1, the structure of HNMT inhibitors differs widely in structure and pharmacologic profile [147].

Although several compounds have demonstrated HNMT inhibition, its effects in increase histamine level its activity is not directly correlated with its ability to increase histamine brain levels due to poor blood-brain penetration such as amodiaquine [148], and quinacrine [149], or has not been explored such as etoprine [150].

In this sense, increased of histamine levels by metoprine after oral administration (10 mg/kg) have been corroborated. Indeed, histamine levels achieved by metroprine have been found to be elevated more than two-fold 4 h after administration of the drug [149]. The biological effects of increasing histamine levels in the brain have been known by employing metoprine, such as antinociception, suppression of energy intake, improvement of cognitive function, antiepileptic effect, and attenuation of methamphetamine-induced behavioral abnormalities [144].

In addition, dimaprit, a histamine H2-agonist, represents a potent HNMT inhibitor. Effects related to the increase of brain levels of histamine have been corroborated, however, only after intracerebroventricularly administration in rats [151].

Interestingly, by employing in vitro studies with human embryonic kidney and recombinant human brain HNMT, it has been demonstrated that tacrine exhibits HNMT inhibitory activity [153].

Due to the increase in the knowledge of the beneficial effects of the brain by histamine, several groups have developed great efforts to search for novel HNMT inhibitors. A computational study performed by Nurhan et al. found that among a series of phytocompounds obtained from *N. sativa* and *C. xanthorrhiza*, a total of eight metabolites (longifolene, (+)-beta-atlantone, humulene epoxide, (−)-beta-curcumene, (E)-caryophyllene, germacrone, (R)-(−)-xanthorrhizol, and (−)-beta-caryophyllene epoxide} showed great affinity to HNMT, thus highlighting the importance pf continuing their evaluation employing experimental studies [154].

In addition, Ichinose et al. evaluated a series of helicene derivatives as HNMT inhibitors. Interestingly, methyl (P)-1,12-dimethyl-6-iodo-5-(trifluoromethanesulfonyloxy)benzo[c]phenanthrene-8-carboxylate shown activity as HNMT inhibitor at μM order by employing in vitro studies [155].

Consequently, the design and development of novel potent and selective HNMT with high BBB permeability are expected to provide new therapeutic approaches as co-therapy for AD patients.

In this sense, computational studies employing molecular crystal structures of HNMT complexed to diverse inhibitors (amodiaquine, metoprine, quinacrine) demonstrate that these compounds bind to their active site. Analysis at the molecular level allows concluding that Phe9, Tyr15, and Phe19 residues located at the N-terminus are essentials to inhibitor binding. Interestingly, the N-terminus exhibit many numbers of conformational changes, thus allowing the binding of inhibitors with diverse chemical structures being principally hydrophobic and rigid groups [156].

### 5.3. Beneficial Effects of Increased Histamine Levels in the Brains of AD Patients

As it was stated previously, AD patients show a great number of alterations related to the histaminergic system such as neurofibrillary degeneration of TMN, low brain histamine levels, low H1R expression in both the frontal and temporal cortex, in addition to [53,54]. Due to the beneficial effects of histamine, the development of compounds that increase histamine levels results in particular interest as novel therapeutics to treat AD [157]. However, it is essential to highlight that most of the experience of the beneficial effects of increasing histamine levels have been obtained from the employment of H3R antagonist/inverse agonist [158].

#### 5.3.1. Effects on Cognitive Functions and Neuroplasticity

The histamine effects in cognitive performance have been widely demonstrated. Although H1R antagonist has shown a decrease in neuroinflammation, it has also been shown to impair cognitive performance. This fact has been reinforced with the findings that H1R knockout mice show dementia-like manifestations significantly associated with decreased neurogenesis [159].

The beneficial effects of thioperamide, an H3R antagonist, in APP/PS1 Tg mice, have been corroborated. Wang et al. found that thioperamide administration improves cognitive function, lowers neuronal damage, and reduces Aβ pathology in APP/PS1 transgenic (Tg) mice. According to their results, the beneficial effect has been achieved by increasing Aβ clearance by favoring autophagy and lysosomal processing [160].

In addition, the histaminergic system controls learning and memory by modifying the ACh release. Bonini et al. reported that intra-hippocampal administration of histamine after non-reinforced retrieval enabled the consolidation of step-down inhibitory avoidance extinction. Interestingly, this facilitation was reproduced by the HNMT inhibitor SKF91488 [153]. Administration of H3-antagonists/inverse agonists in the basolateral amygdala, increase the release of histamine and consequently increases ACh release [161,162], thus highlighting the connection between histaminergic and cholinergic neurotransmission for consolidation of fear memories. This finding has been consistent with recent studies which show increased cholinergic tone and muscarinic neuromodulation in the maintenance of visual working memory [163].

However, in clinical trials, H3R cognitive function improvement in AD patients are not fully corroborated. For example, ABT-288, a selective H3R antagonist/inverse agonist showed an increase in histamine and ACh release in vitro, its efficacy in AD is still debatable [161].

The increase of histamine levels by inhibition of astrocytic HNMT such as metoprine has been corroborated. Indeed, the beneficial effects in cognitive performance by metoprine have been demonstrated in a mouse model of amnesia induced by scopolamine. Interestingly this beneficial effect was reverted by a blockade of H1R [162].

Indeed, it has been demonstrated that histamine plays an important role in the consolidation of recognition memory, which has been considered to be a critical component of human declarative memory [164], highlighting the importance of developing novel HNMT inhibitors to increase histamine levels.

#### 5.3.2. Increase in the Degradation of Extracellular Aβ Insoluble Plaques

As previously mentioned, the increase of Aβ aggregates have been related to neuronal damage. Experiments performed by Fu et al. 2007 showed that histamine is able to prevent neurotoxicity induced by Aβ in rat phaeochromocytoma (PC12) cell culture. Interestingly, this effect was reversed by H2R and H3R antagonists but not by H1R antagonists [165]. In addition, activation of H1R in astrocytes by histamine results in increased activity and expression of Matrix metalloproteinase-9 (MMP-9), resulting in the cleave of oAβ into less toxic monomeric species [10]. Indeed, drugs employed to treat AD could exert their effects in part by reinforcing histaminergic neurotransmission. Tacrine, an inhibitor of AChE, which increases ACh neurotransmission, also inhibits the HNMT, consequently increasing histamine levels in the hippocampus [166].

In addition, it has been shown in vivo that a single administration of memantine, an NMDA antagonist employed to treat mild to severe AD, increases histamine neuron activity and potency of histamine neurotransmission [167].

#### 5.3.3. Increasing Neuronal Survival and Neurogenesis

Astrocytes not only interact with histamine by promoting its clearance by HNMT. Histamine could exert its neuroprotective effect by reducing astrocyte cytokine production and increasing the release of GDNF and Neurotrophin-3 (NT-3) by activating H1R, H2R, and H3R in this cell type [85,167]. Moreover, the release of GDNF by astrocytes due to stimulation by histamine can promote neuronal survival and the maintenance of synaptic homeostasis [168]. In addition, NT-3 promotes neuronal survival and plasticity, important processes to CNS homeostasis [169].

Few studies have focused on elucidating the molecular mechanisms by which histamine stimulates neurogenesis. Cell proliferation is followed by cell differentiation in neurogenesis [165]. Histamine increases neuron proliferation via rospero1 and neurogenin1. Additionally, histamine increases the expression of fibroblast growth factor receptor 1 after the activation of H1R [170]. Proliferation induced by histamine has been related to H2R, while the H1R has been related to the differentiation of neural stem cells [171].

### 5.4. Increasing Histamine Levels Could Be Helpful in Neurodegenerative Diseases

HNMT inhibitors represent an important target to develop novel therapeutic agents for AD patients. Additionally, the pivotal participation of HNMT on histamine degradation in the CNS highlights its therapeutic employment against brain diseases.

In addition, the pharmacological employment of HNMT inhibitors is not restrained to AD, since an impairment of the histaminergic system has been attributed as a causative role in other neurological disorders such as narcolepsy [172], Tourette’s syndrome [173], and depression [174]. Accordingly, the evaluation of HNMT inhibitors in the pathologies mentioned above appears to have therapeutic potential.

### 5.5. Potential Adverse Effects by Increasing Histamine Levels

The peripheral inhibition of HNMT could increase of tissue histamine levels, thus, probably increasing the risk to present allergic rhinitis, urticaria, and gastric ulcers. Interestingly, C314T (Thr105Ile) polymorphisms of the HNMT, which results in low activity, have not been related to allergic asthma and rhinitis [175] or effects in the skin and stomach [176]. Thus, it was expected that adverse effects related to HNMT could be minimal. For this reason, it is of particular interest to develop novel potent HNMT inhibitors to evaluate AD in animal models.

## 6. Conclusions and Future Directions

AD represents a multifactorial neurodegenerative disorder in which a series of neurotransmitter dysfunctions have been reported. Among these, several alterations in the histaminergic system have been reported. Neurofibrillary degeneration of TMN and increase of both H3R and HNMT activity represent the main alterations in the histaminergic system of AD patients, which in turn induce low histamine levels. Thus, the increase of histamine levels appears to be an attractive approach to restore, in part, cognitive functions in AD.

In this sense, HNMT inhibitors could favor beneficial histamine effects in AD brains such as cognitive functions, neuroplasticity, and the degradation of Aβ peptide. In addition, due to the low expected adverse effects of HNMT inhibitors, and its potential beneficial effects in other neurological disorders its of particular interest to develop and evaluate more effective HNMT inhibitors with high BBB penetration in animal models of AD, thus highlighting its relevance.

## Figures and Tables

**Table 1:** Pharmacological characteristics of HNMT inhibitors.

Inhibitor	IC50	Effects on Histamine Brain Levels	Reference
Metoprine 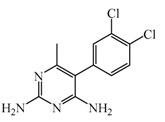	100 nM	Metoprine administered at 10 mg/kg (orally) can increase histamine levels, being the highest 5 h after administration and correlates with peak drug levels in the brain. Histamine levels are still elevated more than 2-fold 4 h after administration of the drug.	[144]
Amodiaquine 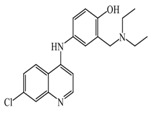	400 nM	Antimalarian agent, potent HNMT inhibitor by in vitro studies. However, amodiquine did not change the endogenous histamine level in the rat brain.	[148]
Quinacrine 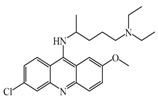	160 nM	Quinacrine, a drug that inhibits HNMT in vitro, has little or no effect on the levels in vivo of histamine.	[149]
Etoprine 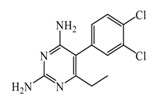	760 nM	Etoprine inhibits dihydrofolate reductase. Etoprine can cross the BBB; however, its effect on histamine brain level has not been explored.	[150]
Dimaprit 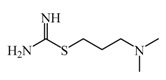	8 μM	Dimaprit, an H2R antagonist, is a potent HNMT inhibitor. Dimatiprit increases histamine brain levels after intracerebroventricularly administration in rats.	[151]
SKF91488 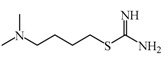	1.85 μM	Due to the low permeability of the BBB SKF91488, research for this compound has been limited.	[152]
